# High-resolution *ex vivo* analysis of the degradation and osseointegration of Mg-xGd implant screws in 3D

**DOI:** 10.1016/j.bioactmat.2021.10.041

**Published:** 2021-11-14

**Authors:** Diana Krüger, Silvia Galli, Berit Zeller-Plumhoff, D.C. Florian Wieland, Niccolò Peruzzi, Björn Wiese, Philipp Heuser, Julian Moosmann, Ann Wennerberg, Regine Willumeit-Römer

**Affiliations:** a Institute of Metallic Biomaterials, Helmholtz-Zentrum Hereon, Max-Planck-Str. 1, Geesthacht, 21502, Germany; b University of Malmö, Faculty of Odontology, Department of Prosthodontics, Carl Gustafs väg 34, Klerken, 20506, Malmö, Sweden; c Lund University, Department of Clinical Sciences Lund, Medical Radiation Physics, Barngatan 4, 22242, Lund, Sweden; d Deutsches Elektronen-Synchrotron (DESY), Notkestrasse 85, 22607, Hamburg, Germany; e Institute of Materials Physics, Helmholtz-Zentrum Hereon, Max-Planck-Str. 1, Geesthacht, 21502, Germany; f University of Gothenburg, Institute of Odontology, Department of Prosthodontics, Medicinaregatan 12 f, 41390, Göteborg, Sweden

**Keywords:** Magnesium alloys, Biodegradable implant, Micro-computed tomography degradation rate, Degradation homogeneity, *Ex vivo* imaging, *Ex vivo* histology, Histology vs. tomography

## Abstract

Biodegradable magnesium (Mg) alloys can revolutionize osteosynthesis, because they have mechanical properties similar to those of the bone, and degrade over time, avoiding the need of removal surgery. However, they are not yet routinely applied because their degradation behavior is not fully understood.

In this study we have investigated and quantified the degradation and osseointegration behavior of two biodegradable Mg alloys based on gadolinium (Gd) at high resolution.

Mg-5Gd and Mg-10Gd screws were inserted in rat tibia for 4, 8 and 12 weeks. Afterward, the degradation rate and degradation homogeneity, as well as bone-to-implant interface, were studied with synchrotron radiation micro computed tomography and histology. Titanium (Ti) and polyether ether ketone (PEEK) were used as controls material to evaluate osseointegration.

Our results showed that Mg-5Gd degraded faster and less homogeneously than Mg-10Gd. Both alloys gradually form a stable degradation layer at the interface and were surrounded by new bone tissue. The results were correlated to *in vitro* data obtained from the same material and shape. The average bone-to-implant contact of the Mg-xGd implants was comparable to that of Ti and higher than for PEEK. The results suggest that both Mg-xGd alloys are suitable as materials for bone implants.

## Highlights

• High-resolution non-destructive synchrotron micro computed tomography of biodegradable Mg alloys *ex vivo*.
• Mg-xGd implants exhibit a high bone-to-implant contact, similar to titanium implants.
• Initially Mg-xGd implants are surrounded by a lesser bone volume fraction but reach similar levels as reference materials.
• Mg-xGd implants degrade at rates lower than 1 mm a^−1^ *in vivo*, decreasing to less than 0.4 mm a^−1^ after 12 weeks.

## Graphical abstract

**Figure:**
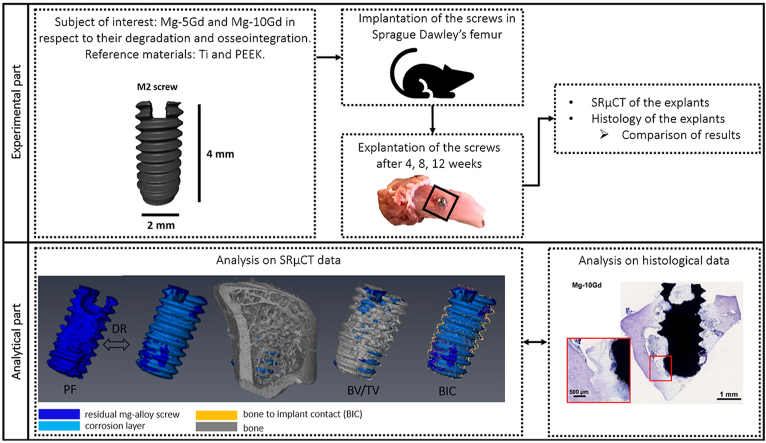

## 1 Introduction

Biodegradable implants emerged as a viable alternative to permanent orthopaedic implants as they eliminate the need for a second surgery to remove the implant, consequently reducing the chance of patients’ harm as well as the financial burden. In comparison to permanent implants, e.g. joint implants, which substitute a missing function and are supposed to stay in the patient for decades, implants meant for osteosynthesis, such as screws, plates or nails, serve for bone support only temporarily [1]. Keeping these implants in the body for longer periods can lead to complications, especially for children, whose growth might be disturbed [1]. Additionally, the modulus of elasticity of conventional permanent implant materials, made of e.g. stainless steel, chrome-cobalt alloys and titanium, is much higher than that of cortical bone. This difference in the elasticity modulus leads to the implant carrying a greater portion of the load and can cause stress shielding effect [2].

Providing an elasticity modulus near to the one of cortical bone [2], being biodegradable and a natural part of the human body, magnesium (Mg) and its alloys are of particular interest as alternative materials for temporary bone implants. The degradation of Mg leads to the formation of harmless corrosion products, which are removed through urine [2,3]. The major limitation of pure Mg is its low corrosion resistance, which can cause a reduction in the mechanical integrity of the implant before the bone or tissue is sufficiently healed. Additionally, a degradation occurring too fast results in the rapid production of hydrogen gas, which then leads to the formation of gas bubbles around the implant. The latter can cause separation of tissue layers from the implant and delays the healing of the tissue (necrosis of surrounding tissue might be the result) [3]. In the worst case, the evolution of gas bubbles may block the blood stream [4]. Another undesirable effect of a rapid degradation is the increase of the pH value in localized areas next to the implant, which can be harmful for the cells [1]. Thus, the degradation rate of the Mg implant must be such that the bone remodeling process occurs before the structural integrity of the implant is compromised.

One way to modulate the corrosion resistance and mechanical properties of Mg is alloying it with other elements. One candidate to improve the corrosion resistance of Mg is gadolinium (Gd), for example added as 5–10 wt percentage (wt. %) to Mg to form alloys [5]. Gd is a rare earth metal (RE), which is already used in medicine as contrast agent. The toxicity of Gd might be a concern, as it can accumulate in animal organs [6]. However, a systematic study on the toxicity and long-term effects of RE elements which are released as ions in the tissue is still missing. For the materials used in this study the release of Gd from Mg-xGd alloys has been shown to be below the toxicity level in cell culture [[7], [8], [9]]. In addition, Mg-10Gd showed in cell culture even an improved osteoblast-induced mineralization [10]. The cells grown on the Mg-10Gd alloy developed healthy cellular structures that allowed them to have good adhesion to the surface [11]. Thus, providing the essential requirements, such as initial mechanical stability, a suitable corrosion rate, and ensuring biocompatibility, Mg-Gd alloys are a possible choice as temporary implant material [12].

In addition to the general biodegradability of the material and its degradation velocity, the degradation homogeneity plays an important role for the performance as an implant material. Mg-based materials undergo galvanic, intergranular, pitting, or crevice corrosion, which all can occur in physiological environments [13,14]. Irregular, localized pitting corrosion may undermine the stability of the implant before the surrounding bone is sufficiently healed [15,16]. Hence, Mg alloys for orthopaedic use should not be subjected to pitting corrosion. To the best of our knowledge, up to now, the pitting behavior of Mg alloys is reported only in *in vitro* studies (e.g. Refs. [17,18]), and often it is only reported for bulk materials, instead that for the final implant design (e.g. Ref. [19]). The description and quantification of the degradation homogeneity of final osteofixation devices, such as pins or screws, is often missing in *ex vivo* or *in vivo* studies.

The *in vitro* degradation behavior of Mg-5Gd and Mg-10Gd screw implants has previously been presented [18]. In that study, Mg-10Gd revealed lower degradation rates, a more homogeneous microstructure degradation performance, and a weaker texture (i.e. orientation of the crystallographic poles in grains in a more random manner, which confers favorable mechanical properties), than Mg-5Gd and could therefore be more suitable as an alloy for load bearing implants. In addition, *Harmuth* et al. showed that it is possible to adjust the mechanical profile of Mg-Gd alloys to the medical requirements by tuning the extrusion process and the Gd content, without influencing the degradation rate [20].

However, the degradation performance of Mg-xGd alloys in bone requires further investigations. A recent publication showed the bone ultrastructure Mg-xGd implants, in comparison with that of more known materials as Ti and PEEK [21]. The bone at the interface with Mg-xGd screws differed significantly from that at the interface with Ti in terms of crystal lattice spacing, suggesting the Mg is potentially incorporated into the bone crystallites during implant degradation and bone healing. However, the crystal lattice spacing between the degradation layer and bone differed, which indicates that Gd potentially remains in the degradation layers and is not incorporated into the bone [21].

In the current work, we aimed to investigate the performance of the aforementioned Mg-xGd alloys with 5 and 10 wt% Gd in bone at high-resolution and over time. To this end, we have employed synchrotron-radiation micro computed tomography (SRμCT). SRμCT is a non-destructive 3D imaging technique with resolutions down to less than 1 μm. The technique enables the simultaneous assessment of the bone, the degraded implant and the degradation layer formed on the surface [22]. Due to the high resolution bone channels are visible, as well as larger secondary phases in the alloy. As the quantitative evaluation of SRμCT is time-consuming and greyscale differences between the degradation layer and bone are low, deep learning techniques can be used to speed up the segmentation process [23,24].

Using SRμCT followed by a segmentation *via* a U-Net convolutional neural network (CNN), we have assessed the bone microstructure and the *in vivo* degradation performance of Mg-xGd alloys. To this end, we implanted Mg-5Gd and Mg-10Gd screws and let them heal for 4, 8 and 12 weeks. After explantation, we studied the degradation rate (*DR*), mean degradation depth (*MDD*), pitting factor (*PF*), bone-to-implant contact (*BIC*) and bone volume fraction (*BV/TV*) and histomorphometrical parameters. Thus, we collected substantial information on the degradation behavior of the chosen materials. Screws of Ti and polyether ether ketone (PEEK) have been implanted as controls. In addition, we compared and correlated the current *ex vivo* results with data on the degradation of the same Mg-xGd alloys obtained previously *in vitro* over 4 and 8 weeks, to understand the prediction capability of *in vitro* testing of the *in vivo* behavior.

## 2 Materials and methods

The production procedure is described in detail in previous publications [18,23]. In brief, permanent mould direct chill casting was used for the melting and casting. The molten materials were then poured into a permanent steel mould. They were solution heat treated (T4) and indirect extrusion was performed with an extrusion ratio of 84 to the final diameter of 12 mm. Rods with a diameter of 3 mm were cut around the center (half radius) of the extruded rods by using wire erosion. The final screw shape was machined by turning and a slit head was formed by milling (4 mm length, 2 mm in diameter, thread M2 and a 0.5 × 0.5 mm slotted screw head). PEEK and Ti screws, purchased from Promimic AB (Mölndal, Sweden), were used as reference materials.

To check the alloys’ homogeneity, the machining quality of the screws before implantation, and for a later comparison with the *ex vivo* results, all Mg-Gd screws were imaged by μCT using a Phoenix Nanotom benchtop μCT (GE inspection and sensing technologies, Wunstorf, Germany) at an operating voltage of 100 kV and a current of 70 μA (binned pixel size: ∼2.5 μm). Screws with large Gd agglomerations or machining defects on the screw surface were discarded.

The Mg screws were cleaned in an ethanol bath, dried, and then packed in individual tubes and thereafter they were gamma-sterilized *via* gamma-irradiation sterilization at a minimum dosage of 27 kGy [25]. The Ti and PEEK screws were cleaned in ethanol bath, dried, then placed in glass vials and autoclaved.

### 2.1 Animal experiments

The animal experiments were conducted after ethical approval by the ethical committee at the Malmö/Lund regional board for animal research, Swedish Board of Agriculture, with the approval number DNR M 188-15. Sixty Sprague Dawley male adult rats with an average weight of 350 g were selected for the study. The rats were housed in cages of 2 or 3 animals each for at least 2 weeks before the beginning of the experiment. The implantation protocol was described in Ref. [21]. In brief, general anesthesia was administered to the rat before starting the surgical procedure and consisted in an intraperitoneal dose of Fentanyl 300 μg/kg + Dexmedetomidin 150 μg/kg. After shaving and disinfection of the rats’ legs (chlorhexidine ethanol solution 0.5 mg/ml, Klorhexidinsprit; Fresenius Kabi, Uppsala Sweden), local anesthetic was injected in the tibial area (1 ml xylocain, Aspen Nordic, Ballerup, Denmark) and a full thickness flap was created. The tibia metaphysis was exposed, and an osteotomy was drilled with a 1.4 mm round bur and then enlarged with a cylindrical bur of 1.6 mm diameter, under constant irrigation with sterile saline. After tapping, the screws were inserted with a manual screwdriver, one in each leg, leaving approximately 2–3 threads of the screw sticking out of the tibial plate, to avoid penetrating the lower cortical bone (monocortical implantation). Each rat received either 2 Mg-based screws (one Mg–10Gd and one Mg–5Gd) or two non-Mg screws (PEEK and Ti), with random allocation to the left and right leg. In total, 30 screws for each material were implanted in 60 rats. After screw insertion, the flaps were sutured. The rats received an analgesic dose of Buprenorfin of 0.01–0.05 mg/kg (Temgesic, Indivior Europe Limited, Dublin, Ireland). The rats were free to move in the cages and were fed *ad libitum*.

After 4, 8, and 12 weeks of healing, the rats were euthanized (20 rats per healing time) with a lethal dose of anesthetic. The legs were dissected, and bone was exposed around the implant area. The implants with bone around were explanted using a trephine bur of 6 mm diameter. The bone-implant blocks were fixed in 70% ethanol for at least 1 day and were then dehydrated in a graded series of ethanol. The samples were critically point dried.

### 2.2 SRμCT data acquisition and analysis

#### 2.2.1 Synchrotron radiation based micro computed tomography (SRμCT)

Imaging of the critically point dried explants was performed at the P05 imaging beamline IBL, which is operated by the Helmholtz-Zentrum Hereon, at the PETRA III storage ring at the Deutsches Elektronen-Synchrotron (DESY) in Hamburg, Germany [26,27]. Samples were scanned during several beamtimes and with various settings due to technical problems with the monochromators and cameras. Various photon energies ranging from 25 to 45 keV were used employing a double crystal monochromator (DCM) or a double multilayer monochromator (DMM). The horizontal beam profile of both monochromators is about 6.5 mm. The vertical beam profile of the DMM is about 7 mm. This is due to an increased vertical divergence of the DMM caused by a slightly bent crystal introduced by its holder. The vertical beam profile of the DCM ranges from about 1.5 to 3.5 mm depending on the X-ray energy. When using the DCM, two height scans were necessary to image the sample. An indirect detector system was used where X-ray photons, which are transmitted by the sample, are converted to optical light by a cadmium tungstate (CdWO4) scintillator screen. The optical light is magnified by a microscope optic and detected by a camera. Two cameras were used: a camera with a CCD (charge-coupled device) sensor (KAF-09000) with 3056 × 3056 pixels, a 16-bit dynamic range and a pixel size of 12.0 μm, and a camera with a CMOS (Complementary metal–oxide–semiconductor) sensor (CMOSIS CMV 20000), which was developed in collaboration with KIT [28], with 5120 × 3840 pixels, a 12-bit dynamic range and a pixel size of 6.4 μm. An objective lens with a fivefold magnification was used resulting in an effective pixel size of 2.4 μm and 1.2 μm, respectively. All samples were imaged using attenuation contrast and a step-wise rotation. For the CCD camera 1200 projections were used. To account for the higher noise and the lower dynamics of the CMOS camera compared to the CCD, a higher number of projections was used from 2400 up to 3000 depending on the lateral extend of the sample and the X-ray energy. Data pre-processing (flat and dark field correction, pixel filtering, beam current normalization) and tomographic reconstruction was implemented in MATLAB (The MathWorks, Inc.) using the tomographic reconstruction pipeline at IBL [29,30]. For tomographic reconstruction the filtered back projection (FBP) algorithm was employed using the ASTRA toolbox for back projection [31,32].

#### 2.2.2 Image segmentation of SRμCT data, registration and resampling of pre-implantation screw

**Fig. 1:**
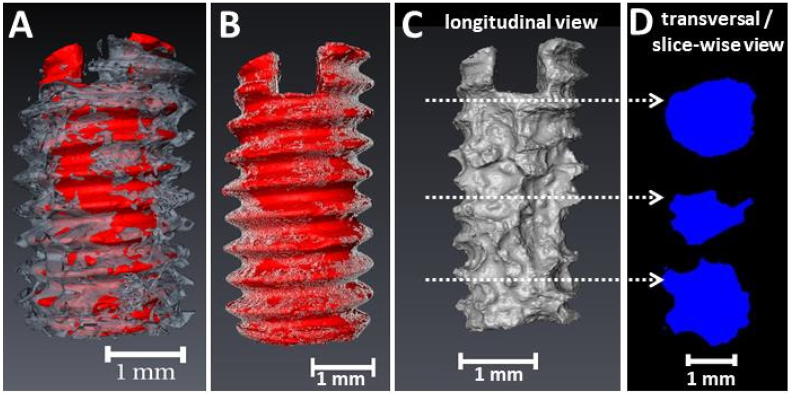
Visualization of a SRμCT scan of a Mg-10Gd implant after 8 weeks *in vivo* using 3D volume renderings (A–C) and cross sections (D). (A) Screw with degradation layer (red) with 200 μm of surrounding bone (grey). (B) Visualization of the BIC (white) over the screw with degradation layer (red). (C) Residual alloy. (D) Transversal cross sections of the residual alloy as indicated in (C). (For interpretation of the references to color in this figure legend, the reader is referred to the Web version of this article).

**Fig. 2:**
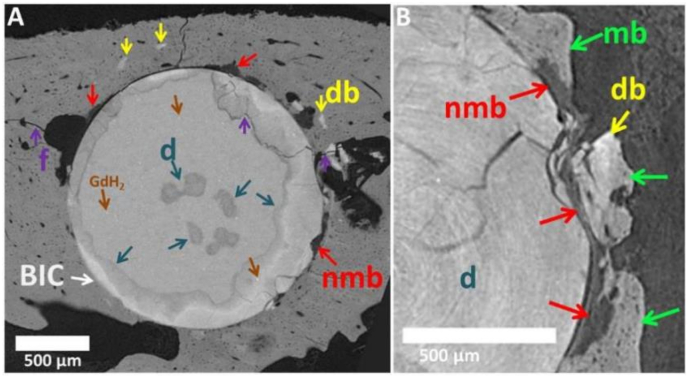
Cross sections of SRμCT scans of two different Mg-10Gd implants after 12 weeks *in vivo* degradation; f = fracture, db = degradation layer in bone, nmb = non-mineralized bone (newly formed bone), mb = mineralized bone, d = degradation layer. Cross-sectional slices of Mg-5Gd, PEEK and Ti can be found in appendices, Fig. 10.The non-mineralized bone is referred as osteoid, because of its very low X-ray attenuation which indicates low levels of Ca. The lacunae and blood vessels are the pores within the mineralized and non-mineralized bone, which differ in size. Lacunae are ellipsoids with sizes smaller than approx. 15 μm. Bigger holes in the mineralized matrix are identified as vessels.

The bone-implant interface exhibits a heterogeneous texture with a linear attenuation coefficient for the degradation layer that varies from the one of bone and the residual alloy, resulting in similar grey values for both materials. Therefore, automatic segmentation approaches failed when applied to the *ex vivo* datasets of the Mg-xGd screws [33]. Due to the fragmented texture of the bone-implant interface, the semi-manual segmentation using a watershed algorithm with an iterative refinement was very cumbersome and time-consuming. Hence, a deep learning based segmentation using a U-net convolutional neural network (CNN) was developed using the hitherto manually segmented annotations as training data [24]. In the segmentation process four labels were used: residual alloy, degradation layer, bone and background. The three-dimensional (3D) volume renderings of the labels are visualized in Fig. 1, while an example cross sectional slice of two Mg-10Gd implants showing the mixture of the materials and tissues with different X-ray attenuation is given in Fig. 2. The denotations used during the analysis process are listed and described in Table 1.

**Table 1:** Explanation of the denotations used during the μCT image analysis.

Name	Explanation
Pre-implantation screw	Volume of the screws prior to implantation measured on Nanotom lab-source μCT (applies only to the Mg-based screws, as the PEEK and Ti screws are expected not to change their shape during time)
Residual alloy	Volume of the screw in the explanted samples that did not corroded during the observation period. Calculated from the visible screw-shape implants without the degradation layer, on the basis of the absorption behavior.
Degradation layer	Corrosion products of Mg alloys in the explanted samples, remaining attached to the original metal, often maintaining the original threaded shape and distinguished from the original metal on the basis of the absorption behavior.
Screw with degradation layer (“degraded screw” or “implant”)	Volume of the residual alloy and the attached corrosion layers, distinguished from the bone and the other surrounding tissues.
Bone	Volume of the mineralized tissue surrounding the implants in the explants.
Background	Volume of the all the remaining materials that were not assigned to the above labels (like soft tissues, air, water, etc.)

#### 2.2.3 Image analysis of SRμCT data

After segmentation of the *ex vivo* image, the “pre-implantation screw” was registered and resampled on the “screw with degradation layer”. All *ex vivo* data were resampled to a voxel size of 5 μm and the longitudinal axis of each screw was aligned parallel to the z-axis of the coordinate system (example of a transverse view in Figs. 1C and 2A) prior to the analysis. Here, a voxel size of 5 μm was chosen to accelerate the data processing. The parameters are investigated as 2D and 3D parameters and are summarized in Table 2 (with definitions following in subsequent sections).

**Table 2:** Analysis parameters of the SRμCT *ex vivo* samples.

Parameter	Dimension of investigation	
	3D	2D
Degradation rate (DR)	✓	-
Mean degradation depth (MDD)	✓	-
Volume loss	-	✓
Pitting factor (PF)	✓	✓
Bone-to-implant contact (BIC)	✓	✓
Bone volume fraction (BV/TV)	✓	-

The 3D calculations consisted in the calculation of the various parameters over the entire volume. The 2D calculations consisted in the calculation of the various parameters on each slice of each data-set and resulted in a statistical mean value and standard deviation for each parameter. The standard deviation of the certain parameter gives statistical information on the parameter's performance along the screw's height. Hence, the 2D calculations are beneficial, since more information with less samples can be gained.

The 2D and 3D calculations are described in details in another publication [18]

##### 2.2.3.1 Degradation rate (*DR*), mean degradation depth (*MDD*), volume loss *(ΔV)* and pitting factor (*PF*)

The degradation rate (*DR*), the mean degradation depth (*MDD*), the volume loss (Δ*V*) and the pitting factor (*PF*) are defined as

(1) $$DR\;\left[mm\;a^{-1}\right]=\frac{\Delta V}{A\cdot t}$$

(2) MDD[μm]=DR⋅t

(3) $$PF=\frac{DP}{MDD}$$

where denotes A the initial surface area, t the degradation time, and *DP* the depth of deepest pit. By fitting a line to the *MDD* values, the slope of the fitted line was calculated and defined as the global degradation rate (*GDR*) in mm a^−1^. Details of the calculations are described in Ref. [18].

##### 2.2.3.2 Bone to implant contact *(BIC*)

In order to evaluate how well the implant is integrated into the bone (osseointegration), in the tomographic data the bone to implant contact *(BIC(t)*, where *t* stands for tomography*)*, which is the contact area of the screw (with the degradation layer) and the surrounding bone, is calculated (see Fig. 1B for the visualization of the *BIC(t)*). The normalized *BIC(t)* is given by:

(4) $$3D–BIC\left(t\right)\;\left[\%\right]=\frac{\#\;surface\;voxels\;of\;implant\;in\;contact\;with\;bone\;}{\#\;total\;surface\;voxels\;of\;ncs}$$

here, the implant stands for the *screw with degradation layer*, defined earlier in Table 1. To determine the contact voxels, the implant layer was dilated once using the image processing package Fiji/ImageJ [34,35] and added to the label bone. The voxels shared by the label “implant” and the label “bone” were defined as in bone-to-implant contact (BIC).

##### 2.2.3.3 Bone volume fraction (*BV/TV*)

In order to evaluate the influence of the materials on the surrounding bone formation and the extent of this influence at different distances from the screw, the bone volume (*BV)* normalized by the total volume (*TV*) was calculated in two different volumes of interest (VOIs) around the screws [36]. To two VOIs were obtained as subvolumes from the entire dataset volume by dilating the pre-implantation registered screw 20 and 40 times, respectively, to obtain a 100 μm (VOI1) and 200 μm (VOI2) volume of interest around each screw. The choice of these VOIs is based on the thread's depth of the M2 screws, which is 250 μm. VOI1 represent a volume in close proximity to the screw surfaces and VOI2 represents the volume inside the threads and around the thread tips (Fig. 1A). The number of voxels occupied by mineralized bone in the VOIs in the bone volume (BV) and is calculated as a ratio over the entire volume of the tissues in the VOIs (*BV/TV*), following the formula:

(5) $$3D-\frac{BV}{TV}\left[\%\right]=\frac{\#\;voxels\;of\;bone\;volume}{\#\;voxels\;of\;total\;volume\;of\;the\;VOI}$$

##### 2.2.3.4 Additional segmentation

Due to the weak contrast between the non-mineralized bone and the background, the segmentation could not automatically discriminate the non-mineralized bone from the background in the SRμCT. Non-mineralized bone, present especially at the shorter healing times, was not segmented as bone, but was instead included in the background layer as the soft tissues, and therefore it was excluded from the calculation of the *BIC* and *BV/TV* parameters.

However, on the SRμCT data it was possible to visually identify newly formed non-mineralized bone by the presence of the osteocytes lacunae, as seen in Fig. 2B [37].

To obviate to this problem and to try to quantify the amount of non-mineralized bone, 3 datasets of the Mg-10Gd after 12 weeks in bone were randomly selected and further segmented semi-automatically *via* a region growing algorithm using Avizo (version 9.4.2, Thermo Fisher Scientific, Waltham, MA), to identify and label the newly-formed non-mineralized bone.

Another aspect that was not possible to segment automatically with the deep learning segmentation method was the fragments of the degradation layer that occurred in some samples and were detached from the implants and integrated into the surrounding tissues (Fig. 2B). For that reason, on the same 3 datasets of Mg-10Gd at 12 weeks of healing, the fragments of the degradation layers were segmented semi-automatically *via* a region growing algorithm using Avizo and quantified.

The additional segmentation was not applied to all the dataset because it was greatly time-consuming.

##### 2.2.3.5 Comparison of *in vitro* results from Ref. [18] with *ex vivo* results from this study

Because it is of great interest to understand the predictive capability of *in vitro* studies on the degradation behavior of Mg alloys in bone, the current data obtained by SRμCT in rats at 4, 8 and 12 weeks were compared to similar SRμCT data obtained from an *in vitro* study on the same alloys (Mg-5Gd and Mg-10Gd) in the same M2 screw shape, observed for 4 and 8 weeks. All the details of the *in vitro* experiment are reported in Ref. [18].

In brief, the correlation between and *ex vivo* and *in vitro* results was done by calculating the ratio between each *ex vivo* parameter and the *in vitro* parameter (*p*_*ex vivo*_/*p*_*in vitro*_) at 4 and 8 weeks (the common observation point between the 2 studies), where *p* is the parameter of the interest plotted on the x-axis. Both for *p*_*ex vivo*_ and *p*_*in*_ *vitro* the mean value of the calculation is taken, since there were different amounts of samples in both experiments.

#### 2.2.4 Histology

After SRμCT imaging, the explants were re-infiltrated in absolute ethanol and then embedded in methyl methacrylate resin by LLS Rowiak LaserLabSolutions GmbH (Hanover, Germany). Each sample was cut in half along the screw longitudinal axis with an Exakt saw. One half of each sample was then prepared for non-decalcified histology with the cutting-grinding technique *ad modum* Donath [38]. Sections of about 40 μm were obtained and stained with a solution of Toluidine Blue-Pyronine Y. The other halves of 36 samples (three per material and time point, randomly selected) were prepared for tartrate-resistant acid phosphatase (TRAP) staining, to identify osteoclasts activity in the proximity of the implants. These halves were laser cut with Tissue Surgeon by LLS Rowiak LaserLabSolutions GmbH (Hanover, Germany). Laser microtomy produced approximately 10 μm thick sections of the bone surrounding the implants (not including the implants but including some degradation layer) mounted on glass slides. The sections were then stained with TRAP using a modification of the protocol described in Ref. [39]. The modified protocol is proprietary by LLS Rowiak (Hanover, Germany). The reagents were purchased from Carl Roth (Karlsruhe, Germany). All stained sections were imaged with a white light optical microscope equipped with a camera (Nikon Eclipse Ci-L and DS-Fi3 camera, Tokyo, Japan). Automatic white-balance was performed on areas of non-tissue background and images were taken at the same exposure conditions. Quantitative histomorphometry was performed using the image analysis software Fiji/ImageJ [34,35] and the parameters defined in the following subsections were calculated.

##### 2.2.4.1 Bone to implant contact (*BIC(h*))

The bone to implant contact from the histology images (*BIC(h),* where *h* stands for histology) was measured on toluidine blue stained sections at 200x magnification. *BIC*(*h*) was calculated as the percentage of the implant perimeter in direct contact with bone, versus the entire implant perimeter inserted in bone (therefore, the parts of the implants sticking out of bone and the implant perimeter inside the screw driver slot were not included in the analysis). For the Mg-based screws, as “screw” was considered the threaded-shaped implant, that included both residual alloy and degradation layers.

##### 2.2.4.2 Bone area (*BA(h*))

Two regions of interests (ROIs) were designed around the screws on toluidine blue stained histological images at 200x magnification. The ROIs included an area of tissue enlarged orthogonally of 100 μm (ROI1) or 200 μm (ROI2) from the perimeter of the screw in each sample. These areas were chosen to describe the influence of the materials on the tissues in the immediate proximity of the implant surfaces (ROI1) and in an area included in the thread depth (ROI2). They overlapped with the selected VOIs.

Bone tissue was segmented from non-bone tissue in the histological images by manual segmentation. The bone area from the histology images (*BA(h)*, where *h* stands for histology) is defined as the area occupied by bone in each ROI, versus the entire area of each ROI. Again, only the area of the implants inserted in the bone was considered for the analysis.

##### 2.2.4.3 Tartrate resistant acid phosphatase (TRAP)-positive area percentages (TRAP%)

This parameter was measured on TRAP-stained sections at 200x magnification. Red-colored pixels were segmented with a color thresholding method in Image J from the non-colored background. The same ROI2 designed for the toluidine blue histological slides was employed also for the TRAP-stained slides. The TRAP-positive area percentage for each sample was calculated as the area stained in red in the ROI2 versus the entire area of ROI2.

#### 2.2.5 Statistical analysis

The mean, median, and standard deviation of all analyzed parameters were calculated for each group. To measure the variation of the pitting factor (*PF*), the volume loss (*volume loss*) and the bone to implant contact (*BIC*) over the entire screw along its longitudinal axis, the coefficient of variation (CV) [40] were calculated. The latter is defined as the ratio of the 2D standard deviation to the 2D mean of the parameter of interest [18]. The *CV PF*, *CV volume loss* and *CV BIC* are used to assess the degradation homogeneity.

Pearson's R correlation (linear correlation) was used to calculate the dependency between the investigated parameters, assuming a normal distribution of the data (in MATLAB R2019b).

Bone-to-implant calculations obtained with histology and SRμCT were compared using a pair Person correlation (a pair being the results of the calculation with the two methods on the same sample). Both *3D BIC(t)* and the mean of *2D BIC(t)* for each sample were compared with the *BIC(h)*.

The mean values of all 2D and 3D parameters for each group (material, time point) were compared using a two-way analysis of variance (ANOVA) multiple comparison test in MATLAB R2019b (The MathWorks Inc., USA) and SPSS Software (IBM, Version 26 USA). Multiple testing correction was performed using the Bonferroni adjustment method [41]. A p-value below 0.05 was considered statistically significant.

## 3 Results

All 60 rats recovered from anesthesia and surgery, and completed the observation period. Nevertheless, not all scanned samples could be properly analyzed after SRμCT due to insufficient image quality and not all samples could be processed for histology, due to technical problems.

The number of analyzed samples for each material and time is presented in Table 3.

**Table 3:** Amount of *ex vivo* samples investigated via SRμCT and histologically.

	SRμCT			Histology		
	4 weeks	8 weeks	12 weeks	4 weeks	8 weeks	12 weeks
Mg-5Gd	9	10	6	10	10	6
Mg-10Gd	8	9	8	7	10	9
Ti	5	8	8	9	9	10
PEEK	9	10	9	10	10	9

### 3.1 Implant degradation velocity and homogeneity from SRμCT analysis

**Fig. 3:**
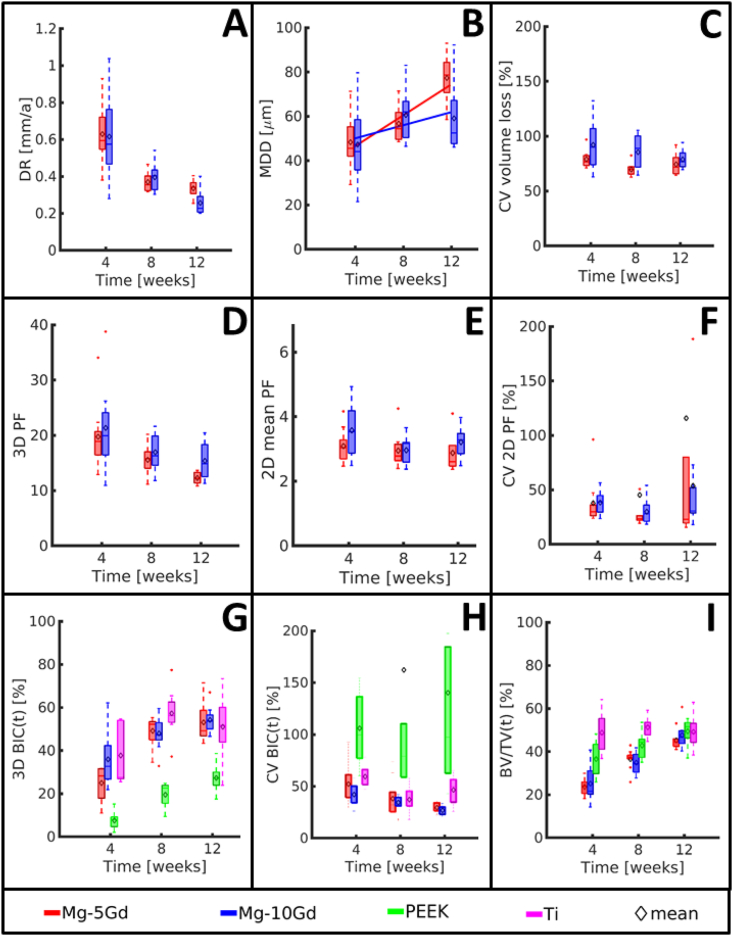
Box plots of the results of the *ex vivo* investigation of Mg-5Gd, Mg-10Gd, PEEK and Ti screw implants over 4, 8, 12 weeks healing period from SRμCT images. (A) Mean degradation depth (*MDD*) with the fitted global degradation rate (*GDR)*, (B) Degradation rate (*DR*), (C) Coefficient of variation of the volume loss (*CV volume loss*), (D) 3D pitting factor (*3D PF*), (E) 2D pitting factor (*2D mean PF*)*,* (F) Coefficient of variation of 2D pitting factor (*CV 2D PF*), (G) 3D Bone implant contact (*3D BIC*), (H) Coefficient of variation of 2D bone implant contact (*CV BIC*), (I) Bone volume density in a 200 μm VOI around the screw surfaces (*BV/TV*). (Middle line of box plot represents the median, ♢ represents the mean, whiskers correspond to 99% confidence interval. See Table 7 and Table 6 for numeric values).

**Fig. 4:**
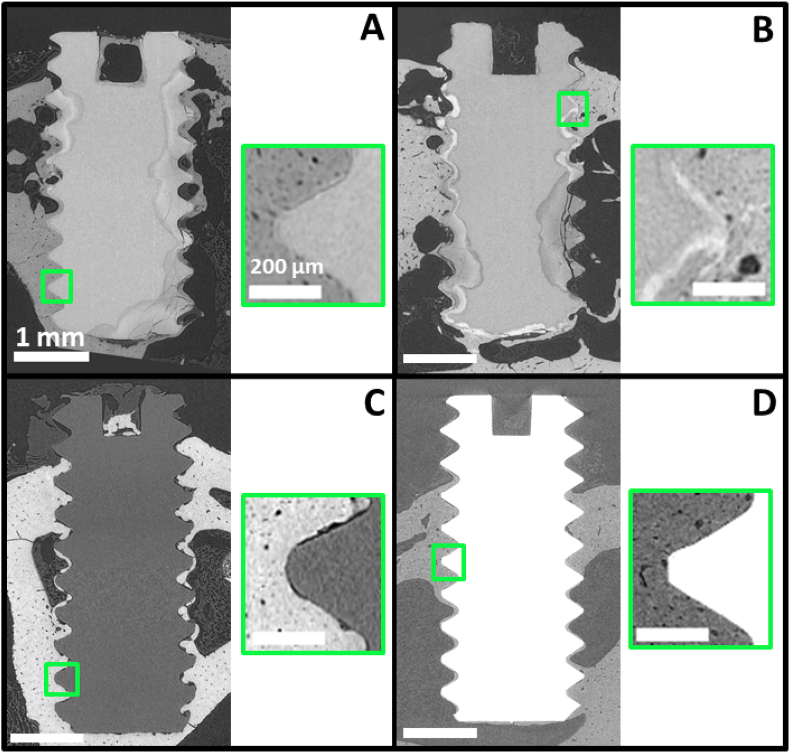
Longitudinal slices of *ex vivo* SRμCT scans 12 weeks after implantation. (A) Mg-10Gd, (B) Mg-5Gd, (C) PEEK, (D) Ti. The image contrast was adjusted for better visualization, which results in the different grey level appearances of the bone in the images. The respective scale bars apply to all images. The thin black space between the bone (whiter) and the PEEK (darker grey) in (C) PEEK is the background, a small gap between the bone and the implant. Such gap is not visible in (A) Mg-10Gd, (B) Mg-5Gd or (D) Ti. This gap for PEEK can be found also in Fig. 10 in appendices.

**Fig. 5:**
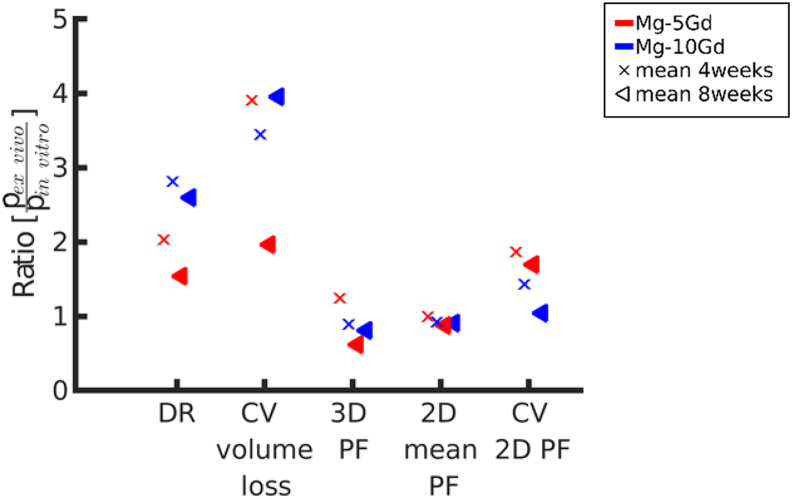
Ratio between mean *ex vivo* and mean *in vitro* analysis parameters (p). The parameters plotted on the x-axis are degradation rate (*DR*), coefficient of variation of the volume loss (*CV volume loss*), 3D pitting factor (*3D PF*), 2D pitting factor (*2D mean PF*) and coefficient of variation of the 2D pitting factor (*CV 2D PF*). The *in vitro* analysis is published in Ref. [18]. The results are summarized in Table 8.

All parameters obtained from SRμCT data are presented in Fig. 3 as box plots, while exemplary longitudinal slices of the bone-implants samples are presented in Fig. 4. All values with details can be found as the supplementary information (Table 6 and Table 7). The significant differences observed are displayed in Table 4. The comparison between the *in vitro* volume loss measurements and *ex vivo* analyses is presented in Fig. 5, and the corresponding values can be found in Table 8.

**Table 4:** P-values; *DR (p < 0.05); ◊MDD (p < 0.05); +CV volume loss (p < 0.05); ○3D PF (p < 0.05); 2D mean PF (p < 0.05: no significant differences at all); CV PF (p < 0.05: no significant differences at all); ˅3D BIC (p < 0.005); □CV BIC (p < 0.05); ●BV/TV 200 μm (p < 0.001).

		Mg-5Gd			Mg-10Gd			PEEK			Ti		
		4	8	12	4	8	12	4	8	12	4	8	12
Mg-5Gd	4												
	8	*˅											
	12	*◊˅●											
Mg-10Gd	4		*+○	*◊●									
	8	*˅											
	12	*˅●			●								
PEEK	4		˅	˅	˅	˅	˅						
	8	●	˅□	˅	●	˅□	˅□						
	12	˅●			●			˅	˅				
Ti	4	●			●			˅					
	8	˅●	●		˅ ●	●		˅●	˅□				
	12	˅●			●			˅	˅

**Table 5:** Strength and direction of the linear correlation between the investigated parameters [29].

P1	P2	Mg-5Gd	Mg-10Gd	PEEK	Ti
*3D BIC(t) [%]*	***DR [mm a***^−1^***]***	MS: −0.76 (p = 0.000)	MS: −0.74 (p = 0.000)		
*3D BIC(t) [%]*	***3D PF [a.u.]***	F: −0.42 (p = 0.033)	P: −0.05 (p = 0.000)		
*DR [mm a*^−1^*]*	***3D PF [a.u.]***	P: 0.15 (p = 0.472)	P: −0.19 (p = 0.355)		
*3D PF [a.u.]*	***3D BV/TV(t) [%]***	MS: −0.60 (p = 0.001)	F: −0.37 (p = 0.065)		
*3D BIC(t) [%]*	***3D BV/TV(t) [%]***	VS: 0.86 (p = 0.000)	MS: 0.75 (p = 0.000)	MS: 0.73 (p = 0.000)	P: 0.07 (p = 0.831)
*BIC(h) [%]*	***3D BIC(t) [%]***	MS: 0.74 (p = 0.000)	M: 0.554 (p = 0.008)	M: 0.56 (p = 0.002)	MS: 0.62 (p = 0.004)
*BIC(h) [%]*	***2D BIC(t) [%]***	MS: 0.74 (p = 0.000)	M: 0.53 (p = 0.01)	P: 0.32 (p = 0.1)	MS: 0.64 (p = 0.003)
*BIC(h) [%]*	***BA [%]***	P: 0.25 (p = 0.21)	P: 0.15 (p = 0.48)	M: 0.49 (p = 0.013)	M: 0.42 (p = 0.025)
*BA [%]*	***3D BV/TV(t) [%]***	M: 0.5 (p = 0.007)	P: 0.28 (p = 0.22)	MS: 0.60 (p = 0.002)	MS: 0.73 (p = 0.000)

Table legend: P1 and P2: parameters correlated; VS = very strong, MS = moderately strong, F = fair, P = poor; value: correlation coefficient; p-value: significance of the correlation; 3D BV/TV and BA values are those in the VOI2 and ROI2, within 200 μm from implant surface. The p-values represent the probability that the correlation between investigated parameters occurs by chance.

#### 3.1.1 Degradation rates

Both alloys revealed *DR*s below 1 mm a^−1^ at all time periods (Fig. 3A). There were no significant differences in the *DR*s of different materials for the same time points.

The *MDD* of the *in vivo* corroded implants is presented in 3B. The fitted lines in 3B represent the *GDR* (see Equation (2)). The *GDR* of Mg-5Gd (0.18 mm a^−1^) is more than twice as high as that of Mg-10Gd (0.077 mm a^−1^).

#### 3.1.2 Coefficient of variation of volume loss (*CV volume loss*)

The coefficients of variation of the volume loss (*CV volume loss*) are presented in Fig. 3C. High values for both alloys indicate a high variability in the amount of the degradation layer in different parts of each implant. This inhomogeneity of the degradation performance can be observed in Fig. 4A where the Mg-10Gd screw displays regions with threads that appeared intact, while in most other region they are degraded entirely. When studying Mg-5Gd and Mg-10Gd alloys, we observe a decreasing tendency of *CV volume loss* over time for both alloys. At all time points, Mg-10Gd showed a higher variation between the *CV* values than Mg-5Gd. The latter further showed smaller values of the variation and mean at 4 and 8 weeks than at 12 weeks. This indicates, that Mg-10Gd degraded more inhomogeneously than Mg-5Gd until the 12-week time point. However, for the *CV volume loss*, no significant differences were found for the different materials at the same time points.

#### 3.1.3 Pitting factor (*PF*)

The 3D pitting factor (*3D PF)*, 2D pitting factor *(2D mean PF),* and coefficient of variation of the 2D pitting factor (*CV 2D PF)* are represented in Fig. 3D–F and Table 6. A tendency for the *3D PF* to decrease over time was observed for both materials. No significant differences of the *3D PF* could be found for the two materials and at the same time points. No significant differences of the *2D mean PD*s and *2D CV PF*s were observed, neither between alloys nor time points, and the values were lower than the *3D PF* values.

### 3.2 Comparison *ex vivo* - *in vitro*

In Fig. 5, the ratios between *ex vivo* and *in vitro* results (*in vitro* results shown in Ref. [18]) for 5 selected parameters are displayed.

The DRs observed *ex vivo* were higher than the *in vitro* ones for the same time points (4 and 8 weeks). The difference is notably higher for Mg-10Gd (2.8 and 2.6 times for 4 and 8 weeks, respectively) than for Mg-5Gd (2 and 1.5 times for 4 and 8 weeks, respectively).

The CV volume loss obtained *ex vivo* is almost 4 times higher compared to the one obtained in *in vitro* experiments, indicating a more inhomogeneous distribution of the degradation layer *ex vivo* (Fig. 5).

The comparison of *ex vivo* and *in vitro* homogeneity behavior reveals that *3D PF*, *2D mean PF* and *CV 2D PF* are similar for Mg-10Gd in both experiments (except *CV 2D PF* after 4 weeks). For Mg-5Gd, the *2D mean PF* are similar in both experiments. The mean ratios of the *CV PF* are between 1 and 2, meaning the *ex vivo* samples show higher overall inhomogeneity than the *in vitro* ones. This indicates that the degradation homogeneity of Mg-10Gd is similar for the *ex vivo* and *in vitro* results.

### 3.3 Implant integration into the bone from SRμCT and histology

#### 3.3.1 Bone-to-implant contact (*BIC*)

The results of the osseointegration analyses of all four implant materials, calculated on tomographic data, are presented in Fig. 3G–I (Table 7 in appendix).

**Fig. 6:**
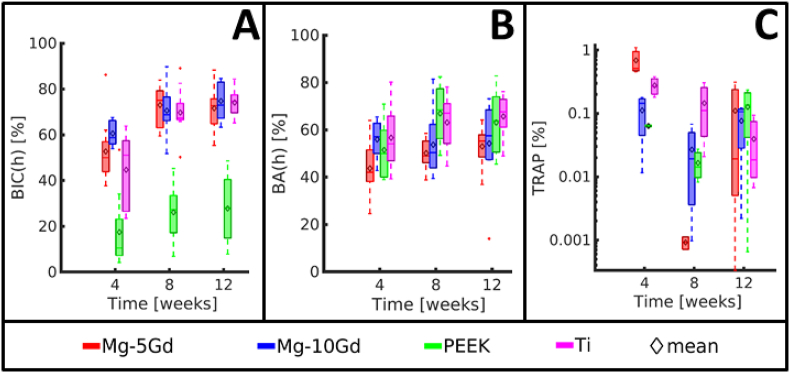
Results of *ex vivo* investigation of Mg-5Gd, Mg-10Gd, PEEK and Ti screw implants over 4, 8, 12 weeks healing period from histological analysis. (A) 2D Bone implant contact (*2D BIC)*, (B) 2D Bone area (*2D BA)* for 200 μm, (C) Tartrate resistant acid phosphatase-positive area (*TRAP*) in a 200 μm ROI around the screw surfaces (Error bars correspond to 99% confidence interval). See Table 7 for numeric values.

All implants showed increasing *3D BIC(t)* over time. Significant differences were found between PEEK and all other materials at all times points. PEEK implants showed on average between 25% and 50% lower *BIC(t)* than the other materials at all time points. This can also be observed in Fig. 6 showing magnifications of a thread for each material. After 12 weeks of implantation, a gap is still visible between the PEEK surface and the surrounding bone, indicating a low osseointegration (Fig. 6C), in contrast to all other materials. Ti implants yielded the highest average *3D BIC(t)* at 4 and 8 weeks. At 12 weeks both Mg alloys reached similar values as Ti, with Mg-10Gd having the highest average *3D BIC(t)* at the longest follow-up time. In general, Mg-5Gd revealed lower (4 weeks) or similar (8 and 12 weeks) *3D BIC(t)* values compared to Mg-10Gd. Both Mg-xGd implants showed smaller variations in *3D BIC(t)* at 12 weeks than the Ti implants.

Since the *2D mean BIC(t)* is nearly equal to the *3D BIC(t)*, it is neither presented in a graph nor discussed. The results of the *CV 2D BIC(t)* are shown in Fig. 3H. The lowest variations were observed in Mg-xGd implants, with Mg-10Gd having slightly lower values than Mg-5Gd. The highest variation was observed in PEEK implants, which can be related to the inhomogeneous distribution of bone-to-implant contact along the implants. Significant differences are found between PEEK after 8 weeks implantation and Mg-10Gd after 8 weeks of implantation. Additionally, a significant difference is observed between PEEK and Ti implants after 8 weeks of healing.

Three Mg-10Gd samples were analyzed after 12 weeks of implantation with respect to the non-mineralized, as well as the mineralized bone. The *3D BIC(t)* excluding the non-mineralized bone resulted 48.1% ± 1.2%, but after adding the non-mineralized bone it became 49.4% ± 1.6%.

**Fig. 7:**
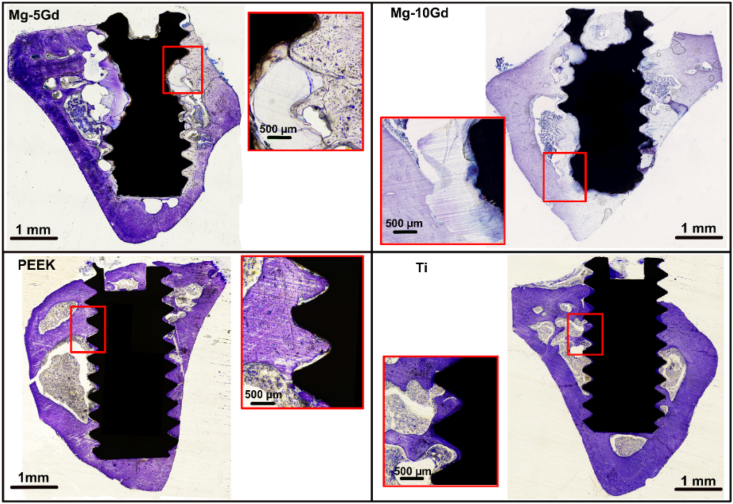
*Ex vivo* histological slices of Mg-5Gd and Mg-10Gd, PEEK and Ti implants after 12 weeks of healing, stained with toluidine blue. (For interpretation of the references to color in this figure legend, the reader is referred to the Web version of this article).

The *BIC(h)* was calculated on histological slides stained with toluidine blue to recognize bone tissue. The results are displayed in Fig. 6A (and in Table 7 in the appendix). Exemplary slices of Ti, PEEK and Mg-5Gd explants can be found in Fig. 7.

For all materials, *BIC(h)* increased from 4 to 8 weeks but remained similar between 8 and 12 weeks. The lowest *BIC(h)* values were observed for PEEK at all time points and the difference was statistically significantly lower for PEEK compared to the other 3 materials at each time point. Both Mg alloys showed higher *BIC* than Ti at 4 weeks with a statistically significant difference between Mg-10Gd and Ti. The average *BIC(h)* values of Mg-5Gd and Mg-10Gd were slightly higher than those of Ti even at 8 and 12 weeks, but at this time point the difference was small and not statistically significant.

#### 3.3.2 Bone volume fraction (*BV/TV(t)*) and bone area (*BA(h)*)

The tendency of the bone volume fraction *BV/TV(t)* was the same for all materials and volumes of interest (VOIs). Therefore, only the results for the 200 μm VOI are discussed and the values for the 100 μm VOIs are presented in supplementary Table 7. The results of the *BV/TV(t)* for VOI2 are presented in Fig. 3I. Ti samples revealed high *BV/TV(t)* values at each time point. In particular, already after 4 weeks of implantation, the amount of bone around Ti implants was close to 50% and remained stable at longer time points. PEEK implants showed a *BV/TV* close to 40% at 4 weeks which increased with time, with the highest average value for all material at 12 weeks (49.4%). At 4 weeks, both Mg alloys showed a *BV/TV* close to 20%, which raised to around 35% at 8 weeks. After 4 and 8 weeks of healing, Mg-xGd implants exhibited significantly lower *BV/TV* values than Ti and PEEK implants. Similarly, for PEEK screws, the average values of *BV/TV* at 4 and 8 weeks were significantly lower than those of Ti. However, at the 12 weeks mark, all materials yielded similar *BV/TV* values, without statistically significant differences (between 45% and 49.3%).

For three Mg-10Gd samples after 12 weeks of *in vivo* degradation the *BV/TV(t)* was calculated including mineralized bone only resulting in a *BV/TV(t)* of 36.6% ± 5.6%, also including non-mineralized bone resulted in a *BV/TV(t)* of 37.6% ± 5.7%.

Bone area *(BA(h))* on the histological slides is the parameter to be compared to the bone volume density (*BV/TV(t))* on tomographic data. It was calculated on two ROIs of 100 and 200 μm from the screw surface. The results of *BA(h)* in the 200 μm ROI are displayed in Fig. 4B (and in Table 7 in the appendix) and discussed below. The results for the other ROI are presented as supplementary material in Table 7.

Ti implants showed a rather stable *BA(h)* over time, between 56.9% ± 13.6% at 4 weeks and 64.8% ± 8.9% at 12 weeks. The *BA(h)* of Ti was significantly higher than that of Mg-5Gd at all three times. The *BA(h)* of PEEK was the one that varied most over time, with an average value of 51.5% ± 11.6% at 4 weeks, which grew to 64.8% ± 11.5% at 8 weeks and 65.26% ± 14.6% at 12 weeks. Both values were the highest of all groups and the difference was statistically significant with Mg-5Gd and Mg-10Gd at 8 weeks, and Mg-5Gd at 12 weeks. The *BA(h)* of Mg-10Gd remained almost constant over time, while that of Mg-5Gd was lower at 4 weeks (44.7% ± 11.4%) and then increased to 53.0% ± 8.6% over time.

#### 3.3.3 Tartrate resistant acid phosphatase (TRAP)-positive area percentages (TRAP%)

The presence of TRAP-positives regions in histological slides represents osteoclasts activated for bone resorption. There was a statistically significant difference in TRAP-positive regions among materials and at different healing times. The highest osteoclast activation for all materials was at 4 weeks, and the difference was statistically significantly higher compared to the other two time points. Then, for Ti sample, it decreased gradually with time, while for Mg-5Gd, Mg-10Gd and PEEK samples, it decreased from 4 to 8 weeks, then slightly increased between 8 and 12 weeks, but the difference between these two points was not statistically significant.

Mg-5Gd slides at 4 weeks showed the highest number of TRAP-positive locations among all materials, statistically significant higher than the other three materials. The differences among Mg-10Gd, PEEK and Ti were not statistically significant at any of the three time points.

**Fig. 8:**
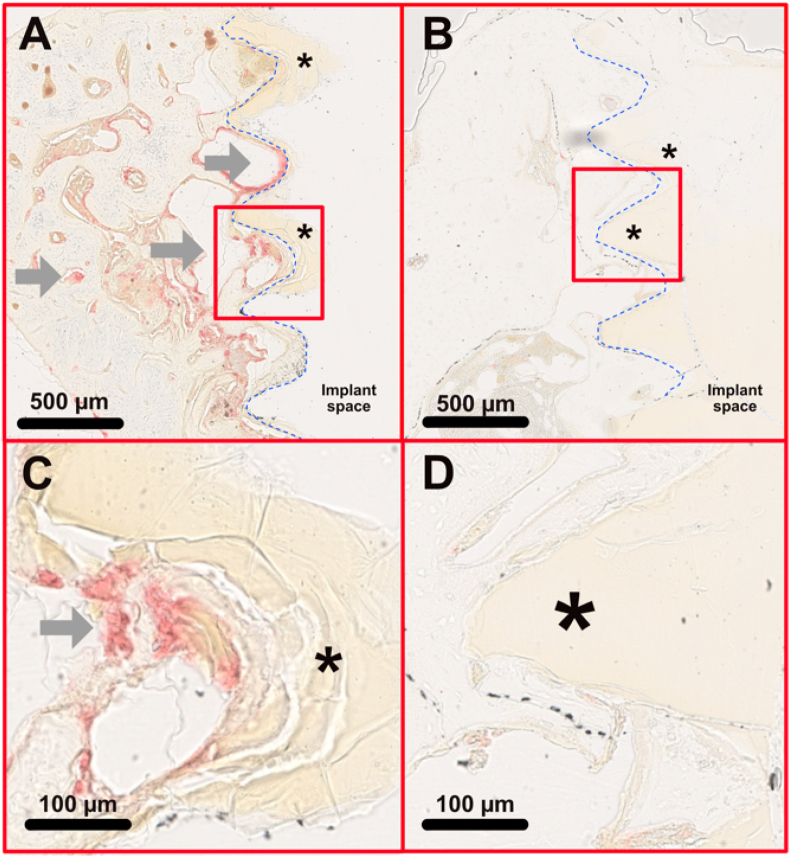
Histological images with TRAP staining of (A) a Mg-5Gd screws after 4 weeks; (B) a Mg-5Gd after 12 weeks of degradation in rat bone. (C) and (D): magnified areas of the above images.

In general, active osteoclasts were concentrated mostly on the surfaces of trabecular bone within the medullar regions of the tibia and less on the cortical regions. Osteoclasts were sporadically found directly on the surfaces of the degradation layer of Mg-5Gd samples (see Fig. 8).

Activated osteoclasts are visible as TRAP-positive pixels (grey arrows). At 4 weeks many TRAP-positive areas are present, especially in the trabecular bone and in regions very close to the implant surfaces. Some active osteoclasts are stained near the degradation layer (asterix). At 12 weeks, very few active osteoclasts could be observed.

### 3.4 Correlations

The results of Pearson's correlation of different parameters are presented inTable 5 with the interpretation of the Pearson's correlation coefficient R from Ref. [42]. Only one very strong correlation between *3D BIC(t)* and *3D BV/TV(t)* was found for Mg-5Gd. This suggests a dependency between the amount of the bone growing in the surrounding of the screw and the bone in direct contact with the Mg-5Gd surfaces. For Mg-10Gd and PEEK the correlation was moderately strong. By contrast, there was no correlation for Ti implants. A moderately strong inverse correlation was found between the *DR* and the *3D BIC(t)* in Mg-5Gd and Mg-10Gd implants. Another moderately strong inverse correlation was found between the *3D PF* and *3D BV/TV(t)* of Mg-5Gd alloy implants. This dependency was just fair for Mg-10Gd. A fair inverse correlation was seen between *3D BIC(t)* and *3D PF* for Mg-5Gd, while there was a very poor, correlation for Mg-10Gd. For both alloys there was a very poor correlation found between *DR* and *3D PF*.

### 3.5 Qualitative observations

#### 3.5.1 Degradation layer behavior

One characteristic aspect of the degraded Mg screws was that they maintained the original threaded shape while they transformed into degradation products. The degradation layers displayed a different grey values range in the tomograms and a different color in the histological slides compared to the original metal. In particular, on histological sections stained with toluidine blue, the degradation layers of Mg-xGd implants were stained in pale purple similarly to the surrounding bone (Fig. 7), but could be distinguished from the bone because they lacked the presence of cells.

**Fig. 9:**
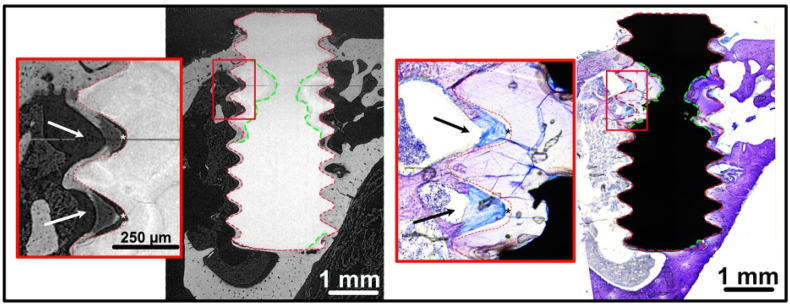
A tomographic slice (left image) and histological slice (right image) of a Mg-10Gd screw after 8 weeks *in vivo*. The green line indicates the border between the residual alloy and the corrosion layer on the histological image. The red line indicates the borders between the corrosion layer and the bone from the tomographic image. The residual alloy appears black in the histology because it is made of the original metal and it does not allow light to pass through. It can be noted that the degradation layers identified on the histological image as part of the screws, but not made of metal, overlap with the same areas identified in the SRμCT as areas with a different absorption coefficient than the residual alloy. Higher magnifications of the μCT slice and of the histology (red square areas) show a region of woven bone, less mineralized. In the histology, the woven bone (black arrows) has a darker colour and rounder osteocites lacunea, compared to the mature bone. In the μCT, the woven bone (white arrows) is less absorbing than the mature bone, probably because it is less mineralized. In addition, this bone is slightly detached from the implant surface (asterixs), probably as an artefact due to the shrinkage occurred to this less mineralized tissue during critical point drying. (For interpretation of the references to color in this figure legend, the reader is referred to the Web version of this article.)

The registration of the tomographic and histological images of the same samples confirmed that what was identified as degradation product on the basis of the grey value, was also stained in purple and identified as degradation layer in the histological images (Fig. 9). This observation validated our segmentation procedure (section 2.2.2).

**Fig. 10:**
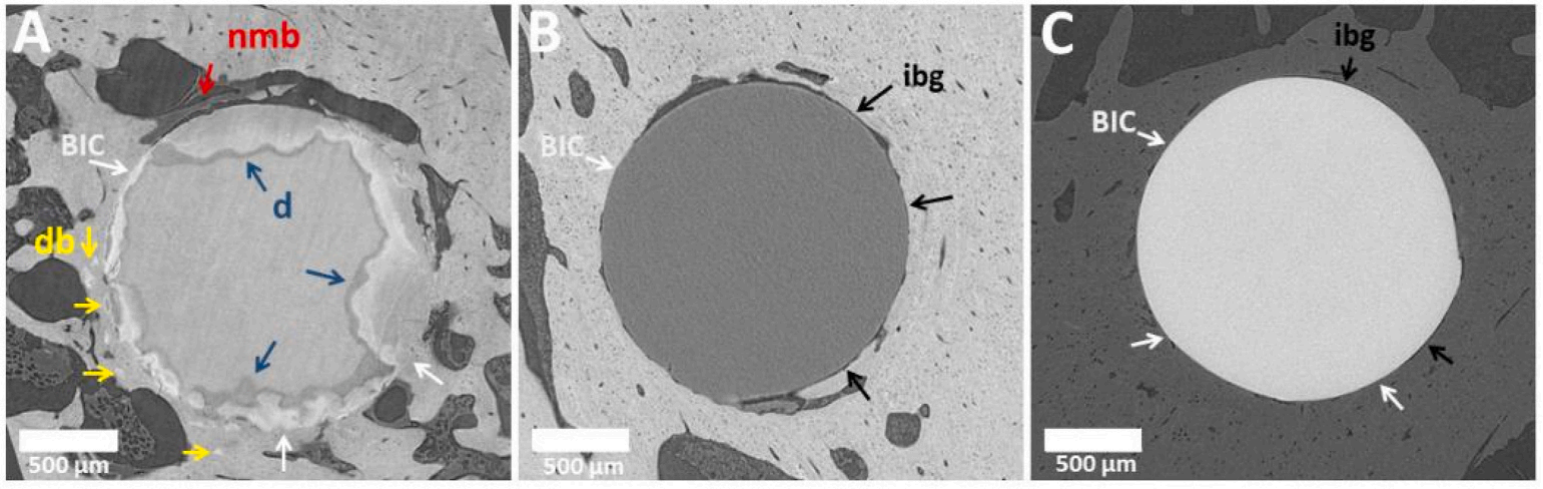
Cross sections of SRμCT scans of implants after 12 weeks *in vivo* degradation;db = degradation layer in bone, nmb = non-mineralized bone (newly formed bone)d = degradation layer; ibg = implant bone gap; (A) Mg-5Gd, (B) PEEK, (C) Ti; The image contrast was adjusted for better visualization, which results in the different grey level appearances of the bone in the images.

Some parts of the degraded screw threads were broken, which might indicate a brittle behavior of the degradation layer (Figs. 2 and 6A and B). Tomograms of three Mg-10Gd screws after 12 weeks healing were visually inspected and segmented with respect to the amount of the degradation layer which was fractured and fully isolated from the screw. Averaged over three samples, 0.62% ± 0.34% of degradation layer was found to be fractured of which 4.6% ± 1.34% were surrounded by bone. The degradation layer debris were often surrounded by mineralized bone and sometimes newly formed non-mineralized bone grew into the fracture line between a detached debris and the implant (Fig. 2B). Cross-sectional SRμCT images of Mg-5Gd, PEEK and Ti as comparison to Fig. 2 after 12 weeks *in vivo* degradation can be found in appendices (Fig. 10).

#### 3.5.2 Tissue integration and tissue reactions around different materials

Bone tissue surrounded the implants of all materials already at 4 weeks, and its amount increased continuous up to week 12. Bone grew into the screw threads, and a thin layer of bone encapsulated most of the screws extending even in those areas that initially were not in contact with bone surfaces (i.e. the medullar space of the tibia). We observed a higher amount of woven bone around the Mg alloys, especially at 4 weeks, while the bone around Ti and PEEK looked more mature. Woven bone was identified by its darker purple/blue color and the presence of larger osteocytes lacunae (magnifications in Fig. 9) with a round shape compared to mature bone that displayed a paler lilac/purple coloration and smaller and almond-shaped osteocyte spaces. Newly formed bone was found predominantly facing regions of the implants that were more degraded, while more mature bone was observed incorporating areas of the implant with less corrosion. Because of the extreme time consumption, quantification of that bone was not possible. The newly formed bone appeared sometimes detached from the surface of the degradation layer, especially in the 4-week samples, but that was likely an artefact of the critical point drying process. Bone was in tight contact with the screw surface of the three metallic materials tested, especially after 12 weeks of healing (Fig. 7). Many of the Mg screws at 12 weeks of healing showed a seamless connection of bone and degradation layers which was hard to distinguish on histological slices, especially considering that the corrosion layer was stained in the same light purple color of the bone (see Fig. 7, in particular the Mg-10Gd screw). Osteocyte lacunae in the bone demarcating Mg materials often appeared to be oriented parallel to the implant surfaces.

In general, no adverse reactions or excessive inflammatory infiltrates were noted around any of the implants in this study.

## 4 Discussion

### 4.1 Degradation behavior

The Mg-10Gd implants in the current study degraded significantly slower than the alloy presented by Galli et al. [43] (ca. 50% and 33% lower after 4 and 12 weeks implantation respectively). However, while the nominal alloys were the same in both studies, other factors were different. The microstructure of the alloy, together with a thorough material characterization, was reported in a recently published paper, in which screws originating from the same batch as those used in the current experiment were examined [18]. In contrast to the screws of Galli et al. [43] the material in Ref. [18] and the current study was not taken from the center of the extruded bar, to avoid stronger galvanic effect by segregation processes from the casting. As one effect, the Gd-rich particles are smaller and more finely distributed in the Mg-xGd alloys of the study in Ref. [18] and the current one.

The study [18] showed that Mg-5Gd and Mg-10Gd had a grain size of 51.78 μm ± 10.91 μm and 26.67 μm ± 1.30 μm, respectively. Therefore, the grain sizes were larger than those reported by *Galli* et al. [43], but the standard deviation among samples were considerably smaller. Additionally, the Gd-rich particles of the alloys in our study were rather fine and homogeneously distributed, as shown in the metallographic examination [18] in contrast to what shown in *Galli* et al. for Mg-10Gd [43]. Another study from *Myrissa* et al. [44] investigated the degradation of Mg-10Gd in the form of pins for 4 weeks in rat femur. The authors reported nearly 0.1 mm a^−1^ slower degradation of the pins, compared to our screws. However, *Myrissa* et al. [44] observed substantial disintegration of the Mg-10Gd pins after 12 weeks in bone, and they could not calculate the degradation rate at that time point because it was not possible to segment all the small remnants of the implants from the bone. In contrast, both alloys in our study appeared manly intact for the entire follow-up time.

The homogeneity of the degradation, which is an important characteristic for the mechanical integrity of the implants during healing, is mainly described by the *PF* with a lower *PF* indicating a more homogenous corrosion behavior. In our study, the *3D PF* was higher for Mg-10Gd than Mg-5Gd and it tended to decrease over time slightly.

One could argue that the pitting corrosion would be mostly visible in the early implantation stages, when the *MDD* values in the *PF* formula are lowest. However, we found a poor correlation between the *DR* and *3D PF* of both alloys, and for that we assumed that, even in the early stages, the degradation homogeneity was not influenced by the higher *DR*s. However, other non-linear correlations could exist between the *DR* and *3D PF*, which were not investigated in this study.

### 4.2 Comparison of *ex vivo* and *in vitro* results

Previous research pointed out that the degradation rate of Mg alloys is usually faster *in vitro* than *in vivo* [44]. However, in our experiments, we observed degradation rates that were higher *in vivo* than *in vitro* (by a factor of 2 and 1.5 for Mg-5Gd and 2.8 and 2.6 for Mg-10Gd, both for 4 and 8 weeks of healing). A similar tendency was observed also in Ref. [43] on Mg-10Gd and Mg-2Ag alloy implants. As discussed in Ref. [43], the reason for this tendency may be that the *in vitro* tests are mostly observed on disks instead than of the actual implants. Moreover, the SRμCT investigations of *ex vivo* data we performed are conducted at a significantly higher resolution than the *in vivo* imaging usually found in literature, which may result in significant differences. A possible explanation for the implants' degradation being faster *in vivo* than *in vitro* could be the influence of the mechanical stresses on the screws either due to the friction of the implant with the osteotomy walls during the insertion or due to the animal's movement during the implantation period in the animal, or a combination of both aspects [43]. Another possible reason for the faster degradation *in vivo* than *in vitro* is that in tissues the pH is continuously buffered and the degradation products as well as Mg ions are quickly removed by the blood flow. Simultaneously new electrolytes are fed to the corrosion sites maintaining the local implantation environment propitious to continuous degradation [45].

We further compared the *PF* observed *in vivo* to the one observed *in vitro* for the same alloys [18]. The *3D PF* was very similar, but slightly lower for Mg-10Gd implants *in vivo* compared to *in vitro* at 4 and 8 weeks. In addition, *3D PF* was lower for Mg-5Gd *in vivo* than *in vitro*. This can be partly explained by the increased *DR in vivo* compared to *in vitro*, since the *PF* is inversely proportional to the *DR*. A higher *DR* can thus result in a smaller *PF* even at equal pits depth in the sample. However, Mg-5Gd implants at 4 weeks of degradation revealed on the contrary higher *3D PF* values *in vivo*, while still having higher *DR*s *in vivo* than *in vitro*. This may indicate that the pitting corrosion for these materials in bone was significant in the early stages of degradation.

The *2D mean PF* was slightly lower *in vivo* than *in vitro*, both at 4 and 8 weeks and for both materials. However, the coefficient of variation (*CV)* of the *2D PF*, which describes the variation between different slices of the screws, was higher *in vivo* than *in vitro*. In addition, we observed a higher *CV* of 2D *volume loss* of the *in vivo* degraded screws for both materials and time points. It revealed a relevant intra-implant variation, because different regions of the same screw corroded differently, but did not display higher pitting factors. In other words, the amount of material degraded is more or less homogeneous when calculated in each slice, without deeper pits, but slices at different heights of the screws showed different amounts of degradation. This is not surprising considering the bone tissue composition in which the screws were immersed. Some parts of the screws were in contact with the hard and compact bone of the cortical region of the tibia. In contrast, other parts were exposed to the trabecular bone with more marrow spaces or even the marrow cavity. Therefore, vascularization and fluid exchange are expected to be different in these regions, providing different environments for corrosion. Moreover, different areas of the screws in contact with the cortical bone of the rat tibia were likely to be exposed to different levels of movement-induced strain, which might have triggered a different degradation behavior. The screws degraded *in vitro* were all immersed in cell culture media and were, therefore, less affected by localized corrosion. Another qualitative observation was that the screws were more degraded at the bottom and at the top. This is likely to be attributed to the larger surface area of the screw which was exposed to the surrounding environment in these regions.

### 4.3 Bone response in relation to degradation

The *BIC,* studied with both imaging methods, tomography and histology, confirmed high values for the Mg materials and Ti and it suggested that both Mg-xGd alloys obtained comparable osseointegration as Ti implants, for the healing times investigated.

The comparisons of *BIC* among different studies should be done with caution, as there is a lot of variability among experiments (e.g. the shape of the implant, the implantation site, thickness of the histological sections as well as the scanning parameters). However, it seems that the current results are in line with other data in the literature. The *3D BIC(t)* of our Mg-xGd materials were similar to the *3D BIC(t)s* of pins made of AZ31 alloy and pure Mg, after 4 weeks of healing in rat bone, but after 12 weeks, Mg-xGd materials showed a lower *3D BIC(t)* of approximately 5% and 20% compared to AZ31 and pure Mg, respectively [46]. However, one reason for that could be that the AZ31 and pure Mg pins were studied with μCT from a lab source, with lower spatial and density resolution, compared to the SRμCT employed here.

Some studies discuss images of bone-implant samples obtained using both methods, showing that laboratory μCT is more prone than SRμCT to artefacts like beam-hardening [47]. Those artefacts affect especially the region of the edge between the metal implant and the bone, making it difficult when not impossible to correctly calculate the *BIC*. Neldam et al. [48] using SRμCT data around Ti implants in bone showed that the *BIC* calculated within 5 μm from the implant was tremendously different than the *BIC* calculated at 50 μm from the implant, arguing that the *BIC* results can vary a lot with the variation of the voxel size. In addition, in many images we found newly formed bone in the proximity of the degradation layers but detached from them and separated by a thin gap, which was completely empty and did not show staining for cells or tissues in histology (Fig. 9). This might be an artefact of the critical point drying procedure. The new bone was probably less mineralized, and it might in fact have shrunk during the drying process, resulting in a thin gap between the bone and the implant. That suggests that the real *BIC* for the Mg alloys was probably higher in the living animal than what we measured *ex vivo*.

One study, employing SRμCT as we did, showed *3D BIC(t)* values for Mg-10Gd screws after 4 and 12 weeks in rats that were 5–10% lower than what we found [43]. However, the *DR* of Mg-10Gd was also higher than what we reported, and that could explain the lower *BIC*, because of the greater surface degradation and formation of hydrogen gas during the early phases of degradation [43].

The *DR* could not be the only parameter that influences hydrogen gas formation and Mg-xGd alloys could have some advantages over pure Mg in that respect. Marco et al. [49] observed that the *DR* (1.11 mm a^−1^ ± 0.05 mm a^−1^) of a Mg-10Gd pin implant was much higher than the one of pure Mg (0.15 mm a^−1^ ± 0.03 mm a^−1^) and Mg-2Ag (0.13 mm a^−1^ ± 0.04 mm a^−1^). However, the number of gas pockets detected with μCT after 7 days post-implantation for Mg-10Gd (0.23 mm^3^ ± 0.32 mm^3^) was much lower than with pure Mg (3.4 mm^3^ ± 1.93 mm^3^) and Mg-2Ag (6.52 mm^3^ ± 8.41 mm^3^). One reason for that is that Mg-RE alloys, such as Mg-10Gd, can absorb hydrogen from the environment and form hydrides in cuboid shape [[50], [51], [52]].

When correlating the *3D BIC* and *DR* of the Mg-xGd alloys, we found a moderately strong inverse correlation. This can be interpreted in two ways. We can hypothesize that when bone is in close contact with the surfaces of the Mg alloys, the *DR* slows down. But, the inverse hypothesis can also be true, which is that when the *DR* is lower, more bone can grow in direct contact with the screws (possibly due to a lower gas formation interfering with the attachment of bone cells to the surface).

Previous *in vivo* investigation utilizing Mg implants have suggested that Mg enhances the bone formation and mineralization compared to permanent implants [[53], [54], [55]] and the possible reasons for that is either the release of Mg ions and/or the alkalization of the local environment during the degradation [43,56,57]. In addition, the degradation layers of Mg materials are rich in calcium and phosphor ions, which are the same components of hydroxyapatite, the bone mineral matrix, and therefore might be osteoconductive [[58], [59], [60]]. In fact, our observation of an increasing amount of *3D BIC(t)* and *3D BV/TV(t)* with ongoing healing time would support the latter suggestion.

Additionally, the fair and poor inverse correlation of *3D BIC* and *3D PF* of Mg-5Gd and Mg-10Gd, respectively, indicate that a relatively high pitting factor does not interfere with the apposition of bone on the implant surfaces. The moderately strong and fair inverse correlation of the *3D PF* and *3D BV/TV* for Mg-5Gd and Mg-10Gd implants could indicate the influence of the surrounding bone on the degradation homogeneity, more so for Mg-5Gd than for Mg-10Gd. Nevertheless, it has to be mentioned that after the elimination of *3D PF* outliers with values larger than 30 for both materials, the correlations assimilated (correlation *3D PF* and *3D BIC*: −0.44 and −0.42 for Mg-5Gd and Mg-10Gd, respectively; correlation *3D PF* and *3D BV/TV*: −0.55 and −0.45 for Mg-5Gd and Mg-10Gd respectively). This could indicate that the performance of both materials is more prone to unexpected and/or different behavior in the beginning of the implantation and is more comparable after a certain period of implantation. However, it is also suggested that in the presence of lower pitting factor there is more bone around the screws. Hence, the homogeneous degradation is in correlation with bone growth and/or the way around - the presence of bone induces homogeneous degradation.

Despite PEEK implants had significantly lower *BIC*s than the other materials, the lowest *BV/TV* was found for Mg-xGd. PEEK displayed a relatively high *BV/TV*s and *BA*s and both values increased with the healing time. This suggested that all materials were gradually surrounded by bone, possibly to shield the implanted material foreign to the body from the richly vascularized medullar cavity. The surrounding bone was in close contact with the three metallic implants including the areas of degradation of the Mg alloys, but it was often slightly detached from the PEEK screws. This finding supports previous observations that PEEK does not promote bone formation when in direct contact with bone, while other implant materials like Ti do [[61], [62], [63], [64]].

The strong and moderately strong correlation found between *3D BIC* and *3D BV/TV* for Mg-5Gd and Mg-10Gd, respectively, suggests that for these materials the presence of more bone tissue in the proximity of the implants increased the probability that this bone was in contact with the implant surfaces. An equally strong correlation was also found for the PEEK implants which displayed overall low *3D BIC* values*.* For Ti, high values of the *3D BIC* and *3D BV/TV* were already observed after 4 weeks of healing. Assuming that the implant stability is correlated to the *3D BIC*, the very poor correlation observed for the Ti implants suggests that the implant stability of Ti screws did not increase over the implantation period.

A high variation of the *BIC* over slices, as described by the *CV BIC*, was observed for the PEEK implants in comparison to the metallic implants. We thus assume a more reliable and more homogeneous osseointegration of the metallic implants.

The formation of new bone in the environment of Mg-10Gd and Ti implants as well as the ingrowth of bone into the degradation layer and the complete encapsulation of the detached debris of the degradation layer by bone for Mg-10Gd material have already been observed qualitatively in Ref. [43]. We confirmed these findings by a quantitative analysis of the distance and the integration of the fractured degradation layer into the bone. We assume that the detachment of small fragments of the degradation layer from the bulk material did not affect the implant stability. The encapsulation of the newly formed degradation layer into bone suggests that the integrity and stability of the Mg-xGd implants did not decrease over time.

Simultaneous to our study, investigations were performed to track the Gd ions from Mg-xGd implants [3]. The main risk with Gd3+ ions is that they have a similar ionic radius to Ca2+, and as such they might be accumulated in the bone. Peruzzi et al. [66] found no traceable amounts of Gd in the bone right next to the implants, neither with EDX nor with neutron tomography. Gd was not detected in the main excretory organs, which might mean that the Gd has remained in the implant site without any interaction. Of course, it could be that there is some Gd mobility, but it is below the detection limits of EDX, neutron tomography or XRF in the organs.

Moreover, using EDX, Peruzzi et al. [66] compared semi-quantitatively the bone in the proximity of the Mg-Gd implants versus the one in the proximity of Ti implants (for exact values Table 1 from Ref. [66]). No Gd was found anywhere, Mg was slightly higher in the bone next to Mg-Gd implants but not high enough to be statistically significant, Ca and P instead were significantly higher next to Ti than next to Mg-Gd. However, Ca and P were basically the same for Mg-5Gd and Mg-10Gd. This means that Mg-Gd implants indeed affects the mineralization of bone in their close proximity, when compared to controls, however a different concentration of Gd does not change this behavior. The latter might suggest, that the main responsible for this effect is probably Mg, rather than Gd.

With respect to the evaluation of the degradation and osseointegration of implants, SRμCT is advantageous over the standard technique of histology, as it allows for a more comprehensive and non-destructive evaluation. In general, SRμCT allows to assess parameters such as the degradation homogeneity or the pitting factor not only on a few, possibly not-representative slices, but throughout the entire sample qualitatively, quantitatively, and in 3D [65].

## 5 Summary and outlook

In the current study, the degradation behavior of two Mg materials containing Gd at different concentrations was investigated using *ex vivo* imaging techniques, SRμCT and histology. The degradation rate and degradation homogeneity were measured quantitatively with good statistics and high-resolution. PEEK and Ti, commonly used in orthopedics, were investigated as reference materials to assess the material-dependent differences in terms of osseointegration using parameters such as the bone-to-implant contact area and the bone volume fraction around the implants.

Acceptable degradation rates and degradation homogeneity of the investigated Mg-xGd materials were confirmed in the current study. The Mg materials displayed comparable osseointegration to the Ti controls. On the other hand, the poor osseointegration of PEEK implants could also be confirmed. A clear assumption about the advantages or disadvantages of any of the investigated Mg-xGd implants could not be made out of the is study's results.

Whether or not the observed high bone-to-implant contact area and bone volume fraction of the Mg-xGd materials are truly indicators for the implant stability needs to be investigated in the future.

## CRediT authorship contribution statement

**Diana Krüger:** Methodology, Investigation, Formal analysis, Software, Writing – original draft, Writing – review & editing. **Silvia Galli:** Investigation, Formal analysis, Writing – original draft, Writing – review & editing. **Berit Zeller-Plumhoff:** Investigation, Supervision, Writing – review & editing. **D.C. Florian Wieland:** Investigation, Supervision, Writing – review & editing. **Niccolò Peruzzi:** Investigation, Writing – review & editing. **Björn Wiese:** Resources, Investigation, Supervision, Writing – review & editing. **Philipp Heuser:** Software, Writing – review & editing. **Julian Moosmann:** Investigation, Supervision, Software, Writing – review & editing. **Ann Wennerberg:** Funding acquisition, Conceptualization, Writing – review & editing. **Regine Willumeit-Römer:** Funding acquisition, Conceptualization, Supervision, Writing – review & editing.

## Declaration of competing interest

The authors declare that they have no known competing financial interests or personal relationships that could have appeared to influence the work reported in this paper.

## Figures and Tables

**Table 6:** Results (mean ± standard deviation) from SRμCT analysis of the *ex vivo* data and the comparison factor between the *ex vivo* and *in vitro* analysis – parameters related to the screw (FM = factor mean, which is the ratio between the mean *ex vivo* value and mean *in vitro* value; FS = factor standard deviation, which is the ratio between the standard deviation *ex vivo* value and standard deviation *in vitro* value).

		4weeks				8weeks				12weeks	
		Mg-5Gd		Mg-10Gd		Mg-5Gd		Mg-10Gd		Mg-5Gd	Mg-10Gd
DR [mm a^−1^]	**FM|FS**	0.63±	2|8	0.62±	2.8|6	0.37±	1.5|5	0.39±	2.6|2.7	0.34±	0.26±
		0.16		0.24		0.05		0.08		0.05	0.07
MDD [μm]	**FM|FS**	48.3±	2|7.5	47.3±	2.8|6	56.7±	1.5|9.2	60.6±	2.6|3.0	77.3±	59.1±
		12		18.3		8.3		12.0		12.0	15.9
CV volume loss [%]	**FM|FS**	79.0 ± 8	3.9|2.5	92.1±	3.4|5.1	69.5±	2|3.6	85.4±	4|2.6	74.4±	78.9±
				23.9		5.4		15.3		10.9	8.4
3D PF	**FM|FS**	19.7 ± 6	1.2|0.5	21.3±	0.9|0.5	15.5±	0.6|2.1	17.0±	0.8|0.2	12.3±	15.4±
				8.4		2.5		3.3		1.1	3.4
2D mean PF	**FM|FS**	3.1 ± 0.5	1|2.5	3.6 ± 0.9	0.9|2.3	2.9 ± 0.5	0.9	3.0 ± 0.4	0.9|1.3	2.9 ± 0.7	3.2 ± 0.5
CV 2D PF [%]	**FM|FS**	38 ± 23	1.9|7.4	38 ± 11	1.4|2.3	45 ± 65	1.7|72.4	30 ± 11	1|2	116 ± 206	54 ± 57

**Table 7:** Results from the SRμCT and histomorphometrical analysis of the *ex vivo* data related to the bone and screw and bone interaction (mean ± standard deviation)

	4weeks				8weeks				12weeks			
	Mg-5Gd	Mg-10Gd	PEEK	Ti	Mg-5Gd	Mg-10Gd	PEEK	Ti	Mg-5Gd	Mg-10Gd	PEEK	Ti
3D BIC [%]	25.0 ± 7.7	36.0 ± 13.0	7.5 ± 3.8	37.7 ± 15.0	49.2 ± 6.3	48.0 ± 7.8	19.5 ± 5.5	57.3 ± 11.5	53.1 ± 10.4	54.3 ± 6.5	27.2 ± 5.8	51.1 ± 14.9
2D BIC mean [%]	25.8 ± 6.9	36.7 ± 13.4	7.2 ± 3.6	36.4 ± 14.3	50 ± 5.9	49.1 ± 7.9	26.4 ± 18	56.7 ± 12.6	54 ± 11.1	55 ± 6.6	34.7 ± 22.5	51.3 ± 14.6
CV BIC [%]	52.3 ± 20.9	42.1 ± 10.8	106.0 ± 36.1	59.3 ± 11.7	38.0 ± 17.1	34.4 ± 7.3	162.3 ± 200.6	37.2 ± 12.3	29.5 ± 5.2	26.8 ± 5.2	140.3 ± 119.7	46.5 ± 14.4
BV/TV [%] - 100 μm	23.5 ± 4.3	25.9 ± 10.1	33.5 ± 6.9	52.3 ± 7.9	40.5 ± 4.2	38.0 ± 4.5	45.5 ± 6.6	59.1 ± 4.6	49.5 ± 5.4	51.5 ± 5.8	59.1 ± 4.6	55.0 ± 6.9
BV/TV [%] - 200 μm	23.6 ± 3.9	25.1 ± 8.5	36.5 ± 7.7	48.8 ± 10.4	36.5 ± 4.3	34.9 ± 5.0	42.8 ± 5.8	51.3 ± 4.6	45.2 ± 4.3	47.6 ± 6.2	49.4 ± 5.7	49.1 ± 7.7
BV/TV [%] - 300 μm	24.8 ± 4.0	26.3 ± 8.2	38.3 ± 7.8	47.3 ± 10.9	35.9 ± 4.5	34.6 ± 5.0	41.3 ± 5.7	47.5 ± 4.3	44.4 ± 3.4	46.7 ± 6.3	47.5 ± 5.4	46.2 ± 7.8
CV BV/TV -200 μm [%]	60.8 ± 6.7	59.0 ± 7.7	98.2 ± 54.1	54.5 ± 9.3	48.0 ± 11.0	43.6 ± 12.7	171.2 ± 201.2	45.1 ± 13.7	36.3 ± 7.0	39.3 ± 7.0	240.1 ± 299.6	55.7 ± 11.1
2D BIC(h) [%]	52.8 ± 13.2	60.5 ± 5.6	17.5 ± 15.1	44.7 ± 16.8	73.0 ± 9.0	70.6 ± 10.3	26.1 ± 12.8	69.7 ± 16.8	71.6 ± 11.1	74.8 ± 8.6	27.8 ± 15.5	74.0 ± 5.7
2D BA(h) [%] – 100 μm	49.8 ± 12	58.6 ± 7.7	52.6 ± 12.8	61.9 ± 11.9	53.5 ± 7.9	59.6 ± 9.3	68 ± 8.2	67.6 ± 10.1	55.9 ± 9	58.5 ± 22.1	68.3 ± 14.6	69.8 ± 6.7
2D BA(h) [%] – 200 μm	43.7 ± 11.2	55.9 ± 8.2	51.5 ± 11.6	56.6 ± 12.2	50.2 ± 6.0	53.7 ± 13.2	67.0 ± 12.4	63.1 ± 11.5	53.0 ± 9.4	54.2 ± 19.0	63.1 ± 14.0	65.6 ± 9.1
TRAP at 200 μm [%]	0.7 ± 0.4	0.1 ± 0.1	0.1 ± 0.0	0.3 ± 0.1	0.0 ± 0.0	0.0 ± 0.0	0.0 ± 0.0	0.1 ± 0.1	0.1 ± 0.2	0.1 ± 0.1	0.1 ± 0.1	0.0 ± 0.0

**Table 8:** Results of different degradation parameters (mean ± standard deviation) of Mg-5Gd and Mg-10Gd screw implants obtained from the *in vitro* experiments, published in Ref. [18] and used for the comparison with the *ex vivo* results from this study

	4weeks		8weeks	
	Mg-5Gd	Mg-10Gd	Mg-5Gd	Mg-10Gd
DR [mm/year]	0.31 ± 0.02	0.22 ± 0.04	0.24 ± 0.01	0.15 ± 0.03
3D PF	15.8 ± 11.8	23.8 ± 15.4	25.1 ± 1.2	20.9 ± 13.3
2D mean PF	3.1 ± 0.2	3.9 ± 0.4	3.3 ± 0.0	3.3 ± 0.3
CV 2D PF [ %]	20.2 ± 3.1	26.7 ± 4.7	26.7 ± 0.9	28.7 ± 5.6
CV dif. layer [%]	20.2 ± 3.1	26.7 ± 4.7	35.3 ± 1.5	21.6 ± 6.0

## References

[1] Willumeit-Römer R. (2019). The interface between degradable Mg and tissue. JOM.

[2] Seal C.K., Vince K., Hodgson M.A. (2009). IOP conference series: materials science and engineering.

[3] Brar H.S., Platt M.O., Sarntinoranont M., Martin P.I., V Manuel M. (2009). Magnesium as a biodegradable and bioabsorbable material for medical implants. Jom.

[4] Kirkland N.T., Birbilis N. (2014).

[5] Hort N. (2010). Magnesium alloys as implant materials--Principles of property design for Mg--RE alloys. Acta Biomater..

[6] Myrissa A., Braeuer S., Martinelli E., Willumeit-Roemer R., Goessler W., Weinberg A.M. (2017). Gadolinium accumulation in organs of Sprague--Dawley®rats after implantation of a biodegradable magnesium-gadolinium alloy. Acta Biomater..

[7] Bruce D.W., Hietbrink B.E., DuBois K.P. (1963). The acute mammalian toxicity of rare earth nitrates and oxides. Toxicol. Appl. Pharmacol..

[8] Feyerabend F. (2010). Evaluation of short-term effects of rare earth and other elements used in magnesium alloys on primary cells and cell lines. Acta Biomater..

[9] Haley T.J., Raymond K., Komesu N., Upham H.C. (1961). Toxicological and pharmacological effects of gadolinium and samarium chlorides. Br. J. Pharmacol. Chemother..

[10] Agha N.A., Willumeit-Römer R., Laipple D., Luthringer B., Feyerabend F. (2016). The degradation interface of magnesium based alloys in direct contact with human primary osteoblast cells. PLoS One.

[11] Cecchinato F. (2015). Influence of magnesium alloy degradation on undifferentiated human cells. PLoS One.

[12] Costantino M.D., Schuster A., Helmholz H., Meyer-Rachner A., Willumeit-Römer R., Luthringer-Feyerabend B.J.C. (2020). Inflammatory response to magnesium-based biodegradable implant materials. Acta Biomater..

[13] Sezer N., Evis Z., Kayhan S.M., Tahmasebifar A., Koç M. (2018). Review of magnesium-based biomaterials and their applications. J. Magnes. Alloy..

[14] Bauer S., Schmuki P., Von Der Mark K., Park J. (2013). Engineering biocompatible implant surfaces: Part I: materials and surfaces. Prog. Mater. Sci..

[15] Zeng R., Dietzel W., Witte F., Hort N., Blawert C. (2008). Progress and challenge for magnesium alloys as biomaterials. Adv. Eng. Mater..

[16] Kirkland N.T., Lespagnol J., Birbilis N., Staiger M.P. (2010). A survey of bio-corrosion rates of magnesium alloys. Corrosion Sci..

[17] Maier P., Zimmermann F., Rinne M., Szakács G., Hort N., Vogt C. (2018). Solid solution treatment on strength and corrosion of biodegradable Mg6Ag wires. Mater. Corros..

[18] Krüger D., Zeller-Plumhoff B., Wiese B., Yi S., Zuber M., Wieland D.C.F., Moosmann J., Willumeit-Römer R. (2021). Assessing the microstructure and in vitro degradation behavior of Mg-xGd screw implants using µCT. J. Magnes. Alloys.

[19] Maier P., Gonzalez J., Peters R., Feyerabend F., Ebel T., Hort N. (2016). Degradation morphology and pitting factor compared to degradation rate. Eur. Cell. Mater..

[20] Harmuth J., Wiese B., Bohlen J., Ebel T., Willumeit-Römer R. (2019). Wide range mechanical customization of Mg-Gd alloys with low degradation rates by extrusion. Front. Mater..

[21] Zeller-Plumhoff B. (2020). Analysis of the bone ultrastructure around biodegradable Mg--xGd implants using small angle X-ray scattering and X-ray diffraction. Acta Biomater..

[22] Zeller-Plumhoff B., Tolnai D., Wolff M., Greving I., Hort N., Willumeit-Römer R. (2021). Utilizing synchrotron radiation for the characterization of biodegradable magnesium alloys—from alloy development to the application as implant material. Adv. Eng. Mater..

[23] Moosmann J. (2017). Developments in X-Ray Tomography XI.

[24] Moosmann J. (2019). Developments in X-Ray Tomography XII.

[25] Feyerabend F., Johannisson M., Liu Z., Willumeit-Römer R. (2015). Influence of various sterilization methods on hardness, grain size and corrosion rate of a Mg6Ag-alloy. BioNanoMaterials.

[26] Haibel A. (2010). Developments in X-Ray Tomography VII.

[27] Wilde F. (2016). AIP conference Proceedings.

[28] Lautner S. (2017). Developments in X-Ray Tomography XI.

[29] Moosmann J. (2014). Time-lapse X-ray phase-contrast microtomography for in vivo imaging and analysis of morphogenesis. Nat. Protoc..

[30] GitHub - moosmann/matlab: data reconstruction and analysis tools for tomography data acquired at the Imaging Beamline (IBL) at P05 and at the High-Energy Material Science (HEMS) beamline at P07 of PETRA III at DESY. https://github.com/moosmann/matlab.

[31] Palenstijn W.J., Batenburg K.J., Sijbers J. (2011). Performance improvements for iterative electron tomography reconstruction using graphics processing units (GPUs). J. Struct. Biol..

[32] Van Aarle W. (2015). The ASTRA Toolbox: a platform for advanced algorithm development in electron tomography. Ultramicroscopy.

[33] Hubbell J.H., Seltzer S.M. (1995).

[34] Schindelin J. (2012). Fiji: an open-source platform for biological-image analysis. Nat. Methods.

[35] Rueden C.T. (2017). ImageJ2: ImageJ for the next generation of scientific image data. BMC Bioinf..

[36] Bouxsein M.L., Boyd S.K., Christiansen B.A., Guldberg R.E., Jepsen K.J., Müller R. (2010). Guidelines for assessment of bone microstructure in rodents using micro--computed tomography. J. Bone Miner. Res..

[37] Griffith J.F., Genant H.K. (2013).

[38] Donath K. (1988).

[39] Erlebacher A., Derynck R. (1996). Increased expression of TGF-beta 2 in osteoblasts results in an osteoporosis-like phenotype. J. Cell Biol..

[40] Abdi H. (2010). Coefficient of variation. Encycl. Res. Des..

[41] Blinowska K.J., Zygierewicz J. (2011).

[42] Chan Y.H. (2003). Biostatistics 104: correlational analysis. Singap. Med. J..

[43] Galli S. (2016).

[44] Myrissa A. (2016). In vitro and in vivo comparison of binary Mg alloys and pure Mg. Mater. Sci. Eng. C.

[45] Wang J. (2015). Recommendation for modifying current cytotoxicity testing standards for biodegradable magnesium-based materials. Acta Biomater..

[46] Kawamura N., Nakao Y., Ishikawa R., Tsuchida D., Iijima M. (2020). Degradation and biocompatibility of AZ31 magnesium alloy implants in vitro and in vivo: a micro-computed tomography study in rats. Materials.

[47] Sarve H. (2011).

[48] Neldam C.A. (2015). Application of high resolution synchrotron micro-CT radiation in dental implant osseointegration. J. Cranio-Maxillofacial Surg..

[49] Marco I. (2017). In vivo and in vitro degradation comparison of pure Mg, Mg-10Gd and Mg-2Ag: a short term study. Eur. Cell. Mater..

[50] Gan W. (2012). Identification of unexpected hydrides in Mg--20 wt% Dy alloy by high-brilliance synchrotron radiation. J. Appl. Crystallogr..

[51] Marco I., Feyerabend F., Willumeit-Römer R., der Biest O. (2016). Degradation testing of Mg alloys in Dulbecco's modified eagle medium: influence of medium sterilization. Mater. Sci. Eng. C.

[52] Peng Q., Huang Y., Meng J., Li Y., Kainer K.U. (2011). Strain induced GdH2 precipitate in Mg--Gd based alloys. Intermetallics.

[53] Jähn K. (2016). Intramedullary Mg2Ag nails augment callus formation during fracture healing in mice. Acta Biomater..

[54] Castellani C. (2011). Bone--implant interface strength and osseointegration: biodegradable magnesium alloy versus standard titanium control. Acta Biomater..

[55] Cheng P. (2016). High-purity magnesium interference screws promote fibrocartilaginous entheses regeneration in the anterior cruciate ligament reconstruction rabbit model via accumulation of BMP-2 and VEGF. Biomaterials.

[56] Bushinsky D.A. (1996). Metabolic alkalosis decreases bone calcium efflux by suppressing osteoclasts and stimulating osteoblasts. Am. J. Physiol. Physiol..

[57] Yoshizawa S., Brown A., Barchowsky A., Sfeir C. (2014). Magnesium ion stimulation of bone marrow stromal cells enhances osteogenic activity, simulating the effect of magnesium alloy degradation. Acta Biomater..

[58] Witte F. (2005). In vivo corrosion of four magnesium alloys and the associated bone response. Biomaterials.

[59] Agha N.A., Feyerabend F., Mihailova B., Heidrich S., Bismayer U., Willumeit-Römer R. (2016). Magnesium degradation influenced by buffering salts in concentrations typical of in vitro and in vivo models. Mater. Sci. Eng. C.

[60] Zeller-Plumhoff B. (2018). Quantitative characterization of degradation processes in situ by means of a bioreactor coupled flow chamber under physiological conditions using time-lapse SRmicroCT. Mater. Corros..

[61] Rao P.J., Pelletier M.H., Walsh W.R., Mobbs R.J. (2014). Spine interbody implants: material selection and modification, functionalization and bioactivation of surfaces to improve osseointegration. Orthop. Surg..

[62] Yoon B.J.V., Xavier F., Walker B.R., Grinberg S., Cammisa F.P., Abjornson C. (2016). Optimizing surface characteristics for cell adhesion and proliferation on titanium plasma spray coatings on polyetheretherketone. Spine J..

[63] Trindade R., Albrektsson T., Galli S., Prgomet Z., Tengvall P., Wennerberg A. (2019). Bone immune response to materials, Part II: copper and polyetheretherketone (PEEK) compared to titanium at 10 and 28 days in rabbit tibia. J. Clin. Med..

[64] Barkarmo S. (2014). Enhanced bone healing around nanohydroxyapatite-coated polyetheretherketone implants: an experimental study in rabbit bone. J. Biomater. Appl..

[65] Tjong W., Nirody J., Burghardt A.J., Carballido-Gamio J., Kazakia G.J. (2014). Structural analysis of cortical porosity applied to HR-pQCT data. Med. Phys..

[66] Peruzzi N. (2021).

